# Extensively-Drug Resistant *Klebsiella pneumoniae* Recovered From Neonatal Sepsis Cases From a Major NICU in Egypt

**DOI:** 10.3389/fmicb.2020.01375

**Published:** 2020-06-19

**Authors:** Noha A. Hassuna, Reem A. AbdelAziz, Amira Zakaria, Mohammed Abdelhakeem

**Affiliations:** ^1^Department of Medical Microbiology and Immunology, Faculty of Medicine, Minia University, Minia, Egypt; ^2^Department of Pediatrics, Faculty of Medicine, Minia University, Minia, Egypt; ^3^Biotechnology Institute, Suez Canal University, Ismaïlia, Egypt; ^4^Department of Clinical Pathology, Faculty of Medicine, Minia University, Minia, Egypt

**Keywords:** *Klebsiella pneumoniae*, PFGE, XDR, NICU = neonatal ICU, sepsis

## Abstract

**Background:**

Neonatal sepsis is a nuisance to clinicians and medical microbiologists, particularly those cases caused by *Klebsiella pneumoniae*. Thus, we aimed at investigating the profile and mechanisms of antibiotic resistance and the clonal relationships between *K. pneumoniae* isolated from neonates at the largest tertiary care hospital’s neonatal intensive care units (NICUs) in Minia, Egypt.

**Methods:**

This study comprised 156 neonates diagnosed with culture-proven sepsis from February 2019 to September 2019, at a major NICU of Minia City. All *K. pneumoniae* isolates were collected and characterized by antimicrobial profile, resistance genotype, and pulsed-field gel electrophoresis typing.

**Results:**

Twenty-four *K. pneumoniae* isolates (15.3%) were collected out of the 156 sepsis diagnosed neonates. These samples showed extensive drug resistance (XDR) to most of the tested antimicrobials, except fluoroquinolones. All the *K. pneumoniae* isolates possessed *bla*_VIM_ and *bla*_NDM_ carbapenemase genes, while *bla*_KPC_ gene was detected in 95.8%. Considering extended-spectrum β-lactamases genes, *bla*_CTX–M_ was found in all the isolates and *bla*_OXA–1_ gene in 75% of them. The plasmid-mediated quinolone resistance gene *qnr*S, was predominantly found among our isolates in comparison to *qnr*B or *qnr*A. A moderate degree of clonal relatedness was observed between the isolates.

**Conclusion:**

To the best of our knowledge, this the first report of an alarming occurrence of XDR among *K. penumoniae* isolates recovered from neonatal sepsis in Egypt. Our data necessitate proper antimicrobial stewardship as the choices will be very limited.

## Introduction

*Klebsiella pneumoniae* is a Gram-negative bacterium, which causes infections with high mortality and morbidity in hospitalized and immunocompromised patients (Gorrie et al., 2017). It is also one of the most frequent causes of outbreaks in neonatal intensive care units (NICUs; Banerjee et al., 2016). This micro-organism has a wide array of resistance mechanisms to several antimicrobials, including beta-lactams, fluoroquinolones, and aminoglycosides (Fair and Tor, 2014; Dsouza et al., 2017). The development of multi-drug resistant *K. pneumoniae* (MDRKP) strains (resistance to three or more antimicrobial agent categories) leads to a growing global burden in choosing appropriate antibiotics in treating hospital-acquired infections (Davies and Davies, 2010). The emergence of MDRKP in healthcare facilities could be attributed to the acquisition of new resistance genes, use of invasive medical devices, inadequate diagnostic and surveillance systems, immunosuppressed states, and inappropriate use of antibiotics (Fletcher, 2015; Ayukekbong et al., 2017; Patolia et al., 2018). Unfortunately, the scenario of antimicrobial resistance has gotten worse in the recent few years, with the appearance of extensively drug-resistant (XDR, sensitive to at most two antimicrobial agent categories), and pan drug-resistant (PDR, resistance to all drugs) *K. pneumoniae* strains (Huang et al., 2018; Krapp et al., 2018). Enormous complications usually follow the infection of these “superbugs” and occasionally death of the infected patients may occur (Giske et al., 2008; Hersh et al., 2012).

There are three main causes of β-lactam antibiotic resistance. First is the production of β-lactamase enzymes by the presence of β-lactam-insensitive cell wall transpeptidases. Second is the active expulsion of β-lactam molecules from Gram-negative bacteria. Third is mutations affecting the expression or function of porins and penicillin-binding proteins (Wilke et al., 2005; Papp-Wallace et al., 2011). Carbapenems are the drugs of choice for treating extended-spectrum beta-lactamase (ESBL)-producing bacterial infections (Karuniawati et al., 2013). Unluckily, there has been an upsurge in carbapenems resistance (World Health Organization, 2017). Plasmid-mediated quinolone resistance (PMQRs) genes: *qnr*A, *qnr*B, *qnr*C, *qnr*D, *qnr*S, and *qnr*VC are accountable for the production of the Qnr proteins. These genes protect bacterial DNA gyrase and topoisomerase IV against quinolones, producing quinolone resistance (Gay et al., 2006).

Neonatal sepsis is the most common cause of morbidity and mortality during the neonatal period. It is classified as early-onset sepsis (EOS; ≤7 days after birth) or late-onset sepsis (LOS; >7 days after birth) (Shah et al., 2015). In NICUs, the rate of sepsis ranges between 28 and 50% of cases with a high prevalence of Klebsiella infections, as bloodstream infections are the major type of infections (Abdel-Wahab et al., 2013). There is a paucity of reports on the prevalence of *K. pneumoniae* in Egypt, especially in NICUs. Accordingly, this study aimed to evaluat the frequency of *K. pneumoniae* among neonatal sepsis cases in a major NICU in Upper Egypt. Specifically, we wanted to determine their antibiotic resistance profile, mechanisms of antimicrobial resistance and clonal relationships, as well as evaluating the clinical outcome of *K. pneumoniae* infected neonates.

## Materials and Methods

### Hospital Setting

Minia University Hospital has a very specialized 20−bed NICU, serving approximately 1200 admissions/year. The hospital has 3500 deliveries/year and operates as a tertiary referral center for neighboring hospitals. The unit consists of two adjacent chambers, including an isolation unit. Neonates comprising premature infants from gestational age (GA) to 23 weeks are managed in the unit.

### Ethics Declaration

In this study, all of the anonymous archival data related to patient age and sex were acquired from the hospital’s archival system. The study was approved by the Ethics Committee in the Faculty of Medicine, Minia University. Consents were obtained from patients’ parents upon sample collection.

#### Subjects

The cohort of this study included neonates who have been admitted to the NICU for more than 48 h. Newborns with evidence of sepsis on admission or within <48 h of admission were excluded from the study. Health-care acquired infection, as defined by Standard Center for Disease Control and Prevention, is an infection that developed during hospital stay (>48 h) by an organism inoculation, which was not found in the patient at the time of admission (Clark et al., 2004). All neonates included in the cohort were attentively assessed throughout their hospital stay for clinical signs of infection. They were followed until hospital discharge or death.

### Study Design

This study was conducted in the period between February 2019 to September 2019. The study included 382 neonates with suspected sepsis according to the Young Infant Study Algorithm (Young Infants Clinical Signs, and Study Group, 2008).

All of the blood samples were taken at the bedside, and then immediately transported to the Microbiology laboratory for further inoculation on suitable culture media and further analysis. Bactec microbial detection system (Bactec 9050, Becton-Dickinson Company, United States) was used for blood cultures. Subcultures were made on blood and MacConkey agar. Isolates were then processed on the VITEK 2 system for identification and antimicrobial susceptibility.

### Phenotypic Identification

The VITEK 2 system (bioMeìrieux, Inc., Hazelwood, MO, United States) was used for the identification of bacteria and determination of their susceptibility pattern according to the Clinical and Laboratory Standards Institute guidelines (CLSI, 2018). *K. pneumoniae* isolates were collected and were further characterized.

The antibiotics used for susceptibility testing were: penicillin derivatives: ampicillin (AMP), β-lactamase inhibitor combinations, Ampicillin/Sulbactam (SAM) and Piperacillin/Tazobactam (TZB); cephalosporins: Cefazolin (KZ), Cefoxitin (FOX), Ceftazidime (CAZ), Ceftriaxone (CRO), and Cefepime (FEP); carbapenems: Meropenem (MEM); aminoglycoside: Amikacin (AMK), Gentamicin (GEN) and Tobramycin (TOB); fluoroquinolone: Ciprofloxacin (CIP) and Levofloxacin (LVX); inhibitors of folate pathway: Nitrofurantoin (F) and Trimethoprim-Sulphamethoxasole (SXT). Isolates were defined as MDR when they were resistant to at least one agent in three or more antibiotic groups (Magiorakos et al., 2012) and as XDR when they were sensitive to at most two antimicrobial agent categories (Huang et al., 2018; Krapp et al., 2018).

### Genomic DNA Extraction

Bacterial DNA was extracted from cultures grown overnight, using the GeneJET genomic DNA purification kit (Thermo Fisher Scientific, MA, United States). Measurement of DNA concentration and purity was done by determining absorbance at wavelengths of 260 nm and 280 nm (NanoDeop1000; Thermo Fisher Scientific). Genomic DNA integrity was confirmed by gel electrophoresis.

### Multidrug Resistance Genes Detection

Polymerase chain reaction (PCR) was used for the detection of the following genes using specific primers: carbapenemases genes (*bla*_VIM_, *bla*_IMP_, *bla*_KPC_, *bla*_NDM_, and *bla*_SPM_), ESBL-encoding genes (*bla*_TEM_, *bla*_SHV_, *bla*_CTX__–__M_ and *bla*_OXA–1_), and PMQRs genes (*qnr*A, *qnr*B, and *qnr*S). Amplification reactions were carried out according to previously described methods (Table 1). For PMQRs, multiplex PCR was carried out using 2 μl of DNA in a total 50 μl reaction mixture having 1× PCR Master Mix (Thermo Fisher Scientific, MA, United States) and 20 pmol of each primer (Cattoir et al., 2007).

**TABLE 1:** Primer sequences that were used in this study.

**Gene**	**5′–3′ sequence**	**Amplicon size**	**References**
*bla*_VIM–1_	5′AGTGGTGAGTATCCGACAG3′	261	Tsakris et al., 2000
	5′ATGAAAGTGCGTGGAGAC3′		
*bla*_IMP–1_	5′CTACCGCAGCAGAGTCTTTG3′	588	Zhao et al., 2009
	5′AACCAGTTTTGCCTTACCAT3′		
*bla*_KPC_	5′ATGTCACTGTATCGCCGTCT3′	893	Bradford et al., 2004
	5′TTTTCAGAGCCTTACTGCCC3′		
*bla*_NDM–_	5′CACCTCATGTTTGAATTCGCC3′	984	Kaase et al., 2011
	5′CTCTGTCACATCGAAATCGC3′		
*bla*_SPM–1_	5′GCGTTTTGTTTGTTGCTC3′	786	Shibata et al., 2003
	5′TTGGGGATGTGAGACTAC3′		
*bla*_TEM_	5′AAACGCTGGTGAAAGTA3′	752	Al-Mayahie, 2013
	5′AGCGATCTGTCTAT3′		
*bla*_SHV_	5′ATGCGTTATATTCGCCTGTG3′	753	
	5′TGCTTTGTTATTCGGGCCAA3′		
*bla*_CTX–M_	5′CGCTTTGCGATGTGCAG3′	551	
	5′ACCGCGATATCGTTGGT3′		
*bla*_oxa–1_	5′ATATCTCTACTGTTGCATCTCC3′	620	
	5′AAACCCTTCAAACCATCC3′		
*qnr*Am	5′AGAGGATTTCTCACGCCAGG3′	580	Cattoir et al., 2007
	5′TGCCAGGCACAGATCTTGAC3′		
*qnr*Bm	5′GGMATHGAAATTCGCCACTGC3′	264	
	5′TTTGCYGYYCGCCAGTCGAAC3′		
*qnr*Sm	5′GCAAGTTCATTGAACAGGGT3′	428	
	5′TCTAAACCGTCGAGTTCGGCG3′		

### Pulsed-Field Gel Electrophoresis

Modified Pulsed Field Gel Electrophoresis (PFGE) Protocol for typing *K.pneumoniae* was used as described by Zakaria and Hassuna (2019). Briefly, 24 *K. pneumoniae* isolates were recovered from −70°C stock culture and streaked on Mueller-Hinton agar (Oxoid, United States). All Culture plates were incubated at 37°C for confluent growth without exceeding a 24-h incubation. Plugs were made by adding an equal volume of molten 1% SeaKem Gold (SKG, Lonza, United States) agarose to the cell suspension, and the mixture was immediately dispensed into the wells of a disposable plug mold (Bio-Rad Laboratories, United States). Plugs were carefully transferred from the mold into new sterile 5 ml plain tubes containing 2 ml lysis buffer. Tubes were incubated overnight at 56°C in shaker incubator with vigorous agitation (170–180) rpm. Plugs were restricted with 50 U Xba1 enzyme (New England Biolabs, United States) and 2 μl BSA (10 mg/ml Thermo Fisher Scientific) and incubated for 16 h at 37°C. Digested slices were loaded into 1% seakem gold agarose with electrophoresis conditions: initial switch time 6.7 s and final switch time 35.6 s and 6 V for 19 h at 14°C. Lambda ladder (New England Biolabs) was used as a molecular size marker.

### Statistical Analysis

The collected data were coded, tabulated, and statistically analyzed using SPSS program (Statistical Package for Social Sciences) software version 25.

Descriptive statistics were done for parametric quantitative data by mean standard deviation and minimum and maximum of the range. For non-parametric quantitative data, statistics were prepared by median and interquartile range (IQR). Statistics were made for categorical data by number and percentage.

Distribution of the data was done by ShapiroWilk test. Analyses were completed for parametric quantitative data between the two groups using Independent Samples *T*-test, and for non-parametric quantitative data between the two groups using Mann Whitney test. Analyses were performed for qualitative data using Fisher’s exact test (more than 20% of cells have an expected count <5). The level of significance was taken at *P*-value < 0.05.

## Results

### Demographic and Clinical Characteristics of the Patients

The number of admitted neonates with suspected sepsis was 382 throughout the study period. Seven cases were excluded due to the loss of five cultures and two were contaminated. Thus, the final number was 375 cases. The incidence of the suspected neonatal sepsis cases amongst the admitted neonates at the NICU was 46.8% (375/860). No growth was detected in 219 blood cultures (58.4%), while bacterial and fungal growth was detected in 156 cases (41.6%).

Since the study focuses on the incidence of *K. pneumoniae*, samples positive for the organism were collected for further characterization. The frequency of *K. pneumoniae* was 15.3%, found in 24 blood cultures from 17 males and 7 females diagnosed with neonatal sepsis. Different presentations on admission included 19 neonates with respiratory distress (79.2%), 2 neonates presented by hypoxic-ischemic encephalopathy (8.3%), another 2 (8.3%) presented by poor suckling and 1 with vomiting (4.2%). Hemoglobin (Hb) levels ranged from 8 to 15 g/dl with mean 10.8 ± 1.8 while white blood cells (WBCs) ranged from 4 to 8 × 10^3^/mm^2^ with mean 5.9 ± 1.5. The rate of neonatal death increased as both weight and gestational age decreased. Platelet count and Hb levels were significantly reduced in cases that eventually died (*P* = 0.005 and 0.045, respectively; Table 2).

**TABLE 2:** Demographic and Laboratory characteristics of the infected neonates with *K. pneumoniae*.

**Characteristic**		**All cases**	**Outcome**		****P**-value**
			**Improved**	**Died**	
		****N** = 24**	****N** = 15**	****N** = 9**	
Weight	Range	(950–4000)	(1500–4000)	(950–3200)	0.002*
	Mean ± SD	2381.3 ± 848.9	2766.7 ± 593.6	1738.9 ± 844	
G.A	Range	(25–38)	(34–38)	(25–38)	0.008*
	Mean ± SD	35.5 ± 3.5	37.3 ± 1.5	32.7 ± 4	
Term	Preterm	10(41.7%)	3(20%)	7(77.8%)	0.009*
	Full-term	14(58.3%)	12(80%)	2(22.2%)	
Onset of sepsis	Early	17(70.8%)	9(60%)	8(88.9%)	0.191
	Late	7(29.2%)	6(40%)	1(11.1%)	
Platelets	Median	40	90	25	0.005*
	IQR	(21.3–112.5)	(40–140)	(15–30)	
Hb	Range	(8–15)	(8–14)	(9–15)	0.045*
	Mean ± SD	10.8 ± 1.8	10.3 ± 1.5	11.8 ± 2	
WBC	Range	(4–8)	(4–8)	(4–8)	0.970
	Mean ± SD	5.9 ± 1.3	5.9 ± 1.4	5.9 ± 1.3	

Abbreviations: GA, gestational age; Hb, hemoglobin; WBC, White blood cells. Independent samples T test for parametric quantitative data between the two groups. Mann Whitney test for non-parametric quantitative data between the two groups. Fisher’s exact test for qualitative data between the two groups. * Significant level at *P*-value < 0.05.

### Antimicrobial Susceptibilities of the Isolates in This Study

**FIGURE 1:**
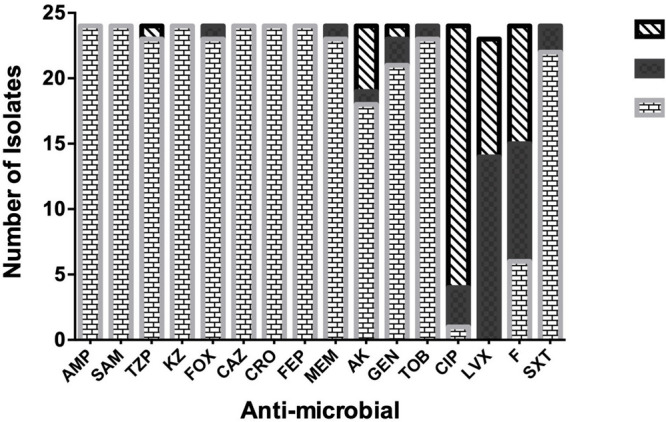
Antimicrobial-susceptibility Profile of neonatal *K. pneumoniae* isolates. Ampicillin: Amp, Ampicillin/Sulbactam: SAM, Piperacillin/Tazobactam: TZB, Cefazolin: KZ, Cefoxitin: FOX, Ceftazidime: CAZ, Ceftriaxone: CRO, Cefepime: FEP, Meropenem: MEM, Amikacin: AMK, Gentamicin: GEN, Tobramycin: TOB, Ciprofloxacin: CIP, Levofloxacin: LVX, Nitrofurantoin: F, Trimethoprim-Sulphamethoxasole: SXT.

Results of the antimicrobial susceptibility testing showed that most of the isolates were XDR (83.3%) and the rest were MDR (17%). All of the isolates (100%) were resistant to AMP, SAM, KZ, CRO, and FEP. Most of them (95.8%) were resistant to FOX, MEM, TZB, and TOB, while none of them were resistant to LVX (Figure 1). The isolates were divided into six resisto-types according to their resistance profile to the eight groups of antibiotics mentioned earlier in the methods section with 0.62 Simpson’s diversity index. The majority of the isolates (83%) had resistance to more than six groups of antimicrobials (Tables 3, 4). No correlation was found between any resisto-type and the clinical outcome of the infected neonates.

**TABLE 3:** Antimicrobial susceptibility pattern and Gene profile of the XDR isolates.

		***qnr*Am**		***qnr*Bm**		***qnr*Sm**		***bla*_TEM_**		***bla*_SHV_**		***bla*_CTX_**		***bla*_OXA_**		***bla*_IMP_**		***bla*_VIM_**		***bla*_SPM_**		***bla*_NDM_**		***bla*_KPC_**	
		**-VE**	**+VE**	**-VE**	**+VE**	**-VE**	**+VE**	**-VE**	**+VE**	**-VE**	**+VE**	**-VE**	**+VE**	**-VE**	**+VE**	**-VE**	**+VE**	**-VE**	**+VE**	**-VE**	**+VE**	**-VE**	**+VE**	**-VE**	**+VE**
Amp	R	24	0	15	9	0	24	23	1	24	0	0	24	6	18	24	0	0	24	24	0	0	24	1	23
A/S	R	24	0	15	9	0	24	23	1	24	0	0	24	6	18	24	0	0	24	24	0	0	24	1	23
Tzp	R	23	0	15	8	0	23	22	1	23	0	0	23	6	17	23	0	0	23	23	0	0	23	1	22
	I	1	0	0	1	0	1	1	0	1	0	0	1	0	1	1	0	0	1	1	0	0	1	0	1
KZ	R	24	0	15	9	0	24	23	1	24	0	0	24	6	18	24	0	0	24	24	0	0	24	1	23
Fox	R	23	0	15	8	0	23	22	1	23	0	0	23	6	17	23	0	0	23	23	0	0	23	1	22
	S	1	0	0	1	0	1	1	0	1	0	0	1	0	1	1	0	0	1	1	0	0	1	0	1
Caz	R	24	0	15	9	0	24	23	1	24	0	0	24	6	18	24	0	0	24	24	0	0	24	1	23
Cax	R	24	0	15	9	0	24	23	1	24	0	0	24	6	18	24	0	0	24	24	0	0	24	1	23
fep	R	24	0	15	9	0	24	23	1	24	0	0	24	6	18	24	0	0	24	24	0	0	24	1	23
Mem	R	23	0	15	8	0	23	22	1	23	0	0	23	6	17	23	0	0	23	23	0	0	23	1	22
	S	1	0	0	1	0	1	1	0	1	0	0	1	0	1	1	0	0	1	1	0	0	1	0	1
Ak	R	18	0	10	8	0	18	17	1	18	0	0	18	5	13	18	0	0	18	18	0	0	18	1	17
	I	5	0	5	0	0	5	5	0	5	0	0	5	1	4	5	0	0	5	5	0	0	5	0	5
	S	1	0	0	1	0	1	1	0	1	0	0	1	0	1	1	0	0	1	1	0	0	1	0	1
Gen	R	21	0	13	8	0	21	20	1	21	0	0	21	5	16	21	0	0	21	21	0	0	21	1	20
	I	1	0	1	0	0	1	1	0	1	0	0	1	1	0	1	0	0	1	1	0	0	1	0	1
	S	2	0	1	1	0	2	2	0	2	0	0	2	0	2	2	0	0	2	2	0	0	2	0	2
Tob	R	23	0	14	9	0	23	22	1	23	0	0	23	5	18	23	0	0	23	23	0	0	23	1	22
	S	1	0	1	0	0	1	1	0	1	0	0	1	1	0	1	0	0	1	1	0	0	1	0	1
Cip	R	1	0	1	0	0	1	1	0	1	0	0	1	1	0	1	0	0	1	1	0	0	1	0	1
	I	20	0	12	8	0	20	19	1	20	0	0	20	5	15	20	0	0	20	20	0	0	20	1	19
	S	3	0	2	1	0	3	3	0	3	0	0	3	0	3	3	0	0	3	3	0	0	3	0	3
LVX	I	10	0	7	3	0	10	9	1	10	0	0	10	3	7	10	0	0	10	10	0	0	10	0	10
	S	14	0	8	6	0	14	14	0	14	0	0	14	3	11	14	0	0	14	14	0	0	14	1	13
F	R	6	0	4	2	0	6	6	0	6	0	0	6	1	5	6	0	0	6	6	0	0	6	0	6
	I	9	0	5	4	0	9	9	0	9	0	0	9	2	7	9	0	0	9	9	0	0	9	1	8
	S	9	0	6	3	0	9	8	1	9	0	0	9	3	6	9	0	0	9	9	0	0	9	0	9
Sxt	R	22	0	13	9	0	22	21	1	22	0	0	22	5	17	22	0	0	22	22	0	0	22	1	21
	I	0	0	0	0	0	0	0	0	0	0	0	0	0	0	0	0	0	0	0	0	0	0	0	0
	S	2	0	2	0	0	2	2	0	2	0	0	2	1	1	2	0	0	2	2	0	0	2	0	2

*Ampicillin: Amp, Ampicillin/Sulbactam: SAM, Piperacillin/Tazobactam: TZB, Cefazolin: KZ, Cefoxitin: FOX, Ceftazidime: CAZ, Ceftriaxone: CRO, Cefepime: FEP, Meropenem: MEM, Amikacin: AMK, Gentamicin: GEN, Tobramycin: TOB, Ciprofloxacin: CIP, Levofloxacin: LVX, Nitrofurantoin: F, Trimethoprim-Sulphamethoxasole: SXT.*

**TABLE 4:** Different Resisto-types of *K. pneumoniae* isolates and its relation to the clinical outcome.

***Resistotype***	**All cases**	**Outcome**		****P**-value**
		**Improved**	**Died**	
	****N** = 24**	****N** = 15**	****N** = 9**	
(A)	7(29.17%)	3(20%)	4(44.4%)	0.179
(B)	13(54.17%)	10(66.7%)	3(33.3%)	
(C)	1(4.17%)	0(0%)	1(11.1%)	
(D)	1(4.17%)	0(0%)	1(11.1%)	
(E)	1(4.17%)	1(6.7%)	0(0%)	
(F)	1(4.17%)	1(6.7%)	0(0%)	

*(A)1,2,3,4,5,7&8, (B)1,2,3,4,5&8, (C)1,2,3,5,8, (D)1,2,3,4,5, (E)1,2,3,4,8, (F)1,2,3,4. 1-Penicillins, 2-β-lactamase inhibitor combinations, 3-Cephalosoprins, 4-Carbapenem, 5-Aminoglycoside, 6-Fluoroquinolone, 7-Nitrofurantoin, 8-Folate-pathway inhibitor. *Simpson’s Diversity index* = 0.62.*

### Genotypic Detection of Carbapenemases, ESBL, and Quinolones Resistance Genes in the Isolates

Regarding the carbapenemases, all the isolates harbored *bla*_VIM_ and *bla*_NDM_ genes, 95.8% possessed *bla*_KPC_ gene, while none of them had either *bla*_IMP_ or *bla*_SPM_ genes. All of the isolates that harbored NDM, VIM, and KPC were resistant to Meropenem, except 1 isolate (Figure 1).

For ESBL genes, 100% of the isolates contained *bla*_CTX–M_, 75% possessed *bla*_OXA–1_ gene, and only 1 isolate had *bla*_TEM_. None of the isolates carried *bla*_SHV_ gene.

All of the 24 isolates (100%) possessed *qnr*S, while 37.5% had *qnr*B and none of them had *qnr*A. QnrS and *qnr*B positive isolates showed a very low level of phenotypic resistance to fluoro-quinolones. Only 1 isolate containing *qnr*S was resistant to ciprofloxacin.

### PFGE

**FIGURE 2:**
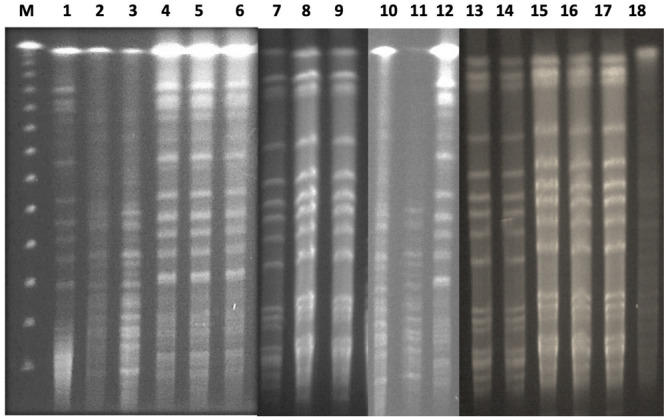
PFGE analysis of clinical neonatal *K. pneumoniae* isolates. Lane 1 (M): lambda PFG ladder marker; lane 2-25: KP isolates 1-24.

**FIGURE 3:**
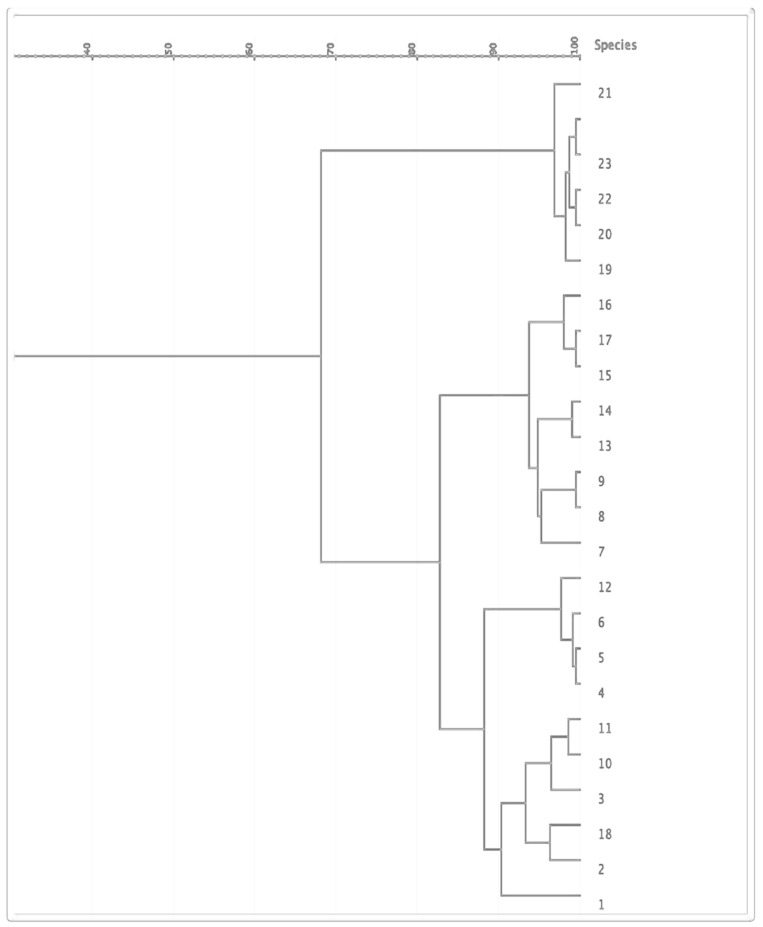
Dendrogram illustrating PFGE patterns of 24 clinical neonatal *K. pneumoniae* isolates.

The 24 isolates were genotyped by PFGE, which showed moderate similarity with 14 pulsotypes presented by more than one isolate (Figure 2). Based on the dendrogram, isolates sharing more than 90% of the bands were included in the same clusters, where they were divided into four main clusters (Figure 3). The resisto-type of each pulsotypes is illustrated in Supplementary Table S1. No significant association was found between different pulsotypes and the presence of various resistance genes (Table 5).

**TABLE 5:** Association between different pulsotypes and the existence of resistance genes.

**Pulsotype**	**No. of isolates**	***qnr*Am**	***qnr*Bm**	***qnr*Sm**	***bla*_TEM_**	***bla*_SHV_**	***bla*_CTX_**	***bla*_OXA_**	***bla*_IMP_**	***bla*_VIM_**	***bla*_SPM_**	***bla*_NDM_**	***bla*_KPC_**
P1	1	0 (0%)	1 (100%)	1 (100%)	0 (0%)	0 (0%)	1 (100%)	1 (100%)	0 (0%)	1100%)	0 (0%)	1 (100%)	1 (100%)
P2	2	0 (0%)	0 (0%)	2 (100%)	0 (0%)	0 (0%)	2 (100%)	1 (50%)	0 (0%)	2 (100%)	0 (0%)	2 (100%)	2 (100%)
P3	3	0 (0%)	1 (33.3%)	3 (100%)	0 (0%)	0 (0%)	3 (100%)	2 (66.7%)	0 (0%)	3 (100%)	0 (0%)	3 (100%)	3 (100%)
P4	3	0 (0%)	1 (33.3%)	3 (100%)	0 (0%)	0 (0%)	3 (100%)	3 (100%)	0 (0%)	3 (100%)	0 (0%)	3 (100%)	2 (66.7%)
P5	1	0 (0%)	0 (0%)	1 (100%)	0 (0%)	0 (0%)	1 (100%)	1 (100%)	0 (0%)	1 (100%)	0 (0%)	1 (100%)	1 (100%)
P6	1	0 (0%)	1 (100%)	1 (100%)	0 (0%)	0 (0%)	1 (100%)	1 (100%)	0 (0%)	1 (100%)	0 (0%)	1 (100%)	1 (100%)
P7	1	0 (0%)	1 (100%)	1 (100%)	0 (0%)	0 (0%)	1 (100%)	0 (0%)	0 (0%)	1 (100%)	0 (0%)	1 (100%)	1 (100%)
P8	2	0 (0%)	0 (0%)	2 (100%)	0 (0%)	0 (0%)	2 (100%)	1 (50%)	0 (0%)	2 (100%)	0 (0%)	2 (100%)	2 (100%)
P9	3	0 (0%)	2 (66.7%)	3 (100%)	0 (0%)	0 (0%)	3 (100%)	3 (100%)	0 (0%)	3 (100%)	0 (0%)	3 (100%)	3 (100%)
P10	1	0 (0%)	1 (100%)	1 (100%)	0 (0%)	0 (0%)	1 (100%)	1 (100%)	0 (0%)	1 (100%)	0 (0%)	1 (100%)	1 (100%)
P11	1	0 (0%)	0 (0%)	1 (100%)	0 (0%)	0 (0%)	1 (100%)	1 (100%)	0 (0%)	1 (100%)	0 (0%)	1 (100%)	1 (100%)
P12	2	0 (0%)	1 (50%)	2 (100%)	1 (50%)	0 (0%)	2 (100%)	2 (100%)	0 (0%)	2 (100%)	0 (0%)	2 (100%)	2 (100%)
P13	1	0 (0%)	0 (0%)	1 (100%)	0 (0%)	0 (0%)	1 (100%)	1 (100%)	0 (0%)	1 (100%)	0 (0%)	1 (100%)	1 (100%)
P14	2	0 (0%)	0 (0%)	2 (100%)	0 (0%)	0 (0%)	2 (100%)	0 (0%)	0 (0%)	2 (100%)	0 (0%)	2 (100%)	2 (100%)
*P*-value		1	0.609	1	0.625	1	1	0.340	1	1	1	1	1

*Fisher’s exact test for qualitative.*

## Discussion

The current study presents the epidemiology, clinical features, outcomes, antimicrobial profile, and molecular basis involved in the possession of XDR *K. pneumoniae* in NICUs in our hospital. The incidence of suspected neonatal sepsis was 46.8% with *K. pneumoniae* being isolated in 15.3% of cases. These results are comparable to studies carried out in Egypt by Abdel-Hady et al. and El-Din et al. (Abdel-Hady et al., 2008; El-Din et al., 2015). On the other hand, higher frequencies of *K. pneumoniae* infection were previously reported in different studies in the same region (Mohsen et al., 2017; Ghaith et al., 2019).

Diagnosis and controlling sepsis face extraordinary challenges as the signs and symptoms are undefined. Additionally, prolonged turnaround time of laboratory diagnosis adds more challenges, which result in the constant prescription of empirical antimicrobial therapy untill the elimination of the suspected sepsis. This results in the emergence of MDR and XDR bacteria, reducing the treatment options, and interfering with efficient treatment. In our NICU, the first-line empirical treatment is ampicillin in combination with gentamicin. If there is no improvement, ampicillin-sulbactam plus a third-generation cephalosporin are used as a second line. This is followed by carbapenems as a third line until the desired blood culture outcomes are received.

Often, XDR strains develop in ICU units as a result of long-term and excessive use of diverse antibiotics (Maseda et al., 2014). The worldwide spread and the rising prevalence of these XDR strains in clinical settings grimly impend public health (Bi et al., 2017). We reported here an alarming occurrence of XDR *K. pneumoniae* in neonatal HCA sepsis with an incidence of 83.3% and resistance to most of the tested antimicrobials except 1 or 2 antimicrobials. This is the first report in Egypt of such resistance rates of *K. pneumoniae* among neonatal sepsis isolates. Previous reports in Egypt showed high resistance rates to ampicillin and cephalosporins, however, susceptibility rates to carbapenems and quinolones were higher (Moore et al., 2005; Fahmey, 2013; El-Din et al., 2015). This high resistance to ampicillin and cephalosporin is due to the use of these antimicrobials as the first and second lines of empirical treatment of neonatal sepsis in Egypt. Resistance to aminoglycoside was also very high in our study, reaching more than 95% of the isolates. This is greater than previously reported by El-Din et al. (2015), who reported an intermediate effect of aminoglycosides (El-Din et al., 2015). The difference in the sensitivity rates to aminoglycoside could be attributed to the difference in the type of the used aminoglycoside. Muhammad and colleagues (Muhammad et al., 2010) found a higher susceptibility to amikacin compared to gentamicin, as the latter was routinely used in the tested NICUs in their study.

Furthermore, a very high frequency of resistance to carbapenems was detected in this study with a rate higher than 95%. This resistance rate is greater than the study performed by Ghaith et al. (2019), who found relatively lower frequencies of carbapenem-resistant *K. pneumoniae* (CRKP) between 43 and 56% in the same country. This difference is probably due to an increase in porin loss, leading to increased carbapenem resistance (Wassef et al., 2015).

The most effective antimicrobial was levofloxacin, with 100% susceptibility, which is in agreement with Mohsen et al. (2017). Although this high susceptibility rate is good news, quinolones are not recommended for use in infants and neonates. However, they might be used in similar cases to ours, where culture-proven sepsis with resistant bacteria exists (El-Din et al., 2015).

Since the second and third lines of treatment in our NICUs depend on the use of cephalosporins as well as carbapenems and that’s why we focused on evaluating the presence of most of the genes responsible for resistance for such antimicrobials. On screening of carbapenemase genes by PCR, all of our isolates harbored *bla*_VIM_ and *bla*_NDM_ genes and most of them possessed *bla*_KPC_ gene. The presence of these genes indicates that carbapenemases are the main mechanism responsible for the presence of CRKP. Our genotypic profile is different from Ghaith et al. (2019), who found a predominance of *bla*_OXA__–48_ (60.8%), while *bla*_NDM_ was found in 52.2% of the isolates and none of their isolates showed *bla*_VIM_. Furthermore, the frequency of *bla*_KPC_ was greater in our study than that found in *K. pneumoniae* isolated in several Egyptian studies (Abdulall et al., 2018; El-Kholy et al., 2018; Ghaith et al., 2019). None of the isolates in this study showed *bla*_IMP_, which does not concur with Melke et al. (Melake et al., 2016), who found it in more than 40% of the isolates obtained from adult ICU patients. On the other hand, the absence of *bla*_SPM_ is in agreement with different studies that reported the absence or rarity of this gene in Egyptian *K. pneumoniae* isolates (Mohamed et al., 2016; Fanaki et al., 2018).

Antimicrobial resistance of *K. pneumoniae* is concomitant mainly with ESBL production. The World Health Organization added (in 2017) ESBL-producing *K. pneumoniae* to the list of the most threatening superbugs together with *Pseudomonas aeruginosa* and *Acinetobacter baumannii* (Branswell, 2017). Concerning ESBL genes in our study, all of the isolates had *bla*_CTX–M_ and 75% had *bla*_OXA–1_ gene. This alarmingly high frequency of ESBL genes is greater than a previous study in Egypt (Abdel-Hady et al., 2008) done on isolates from neonatal sepsis. This increase in ESBL genes detection is probably attributed to the continued prescription of cephalosporins as an essential treatment option in most neonatal sepsis cases. However, it is in concurrence with a study by Amer et al. (Amer et al., 2020), who reported similar rates, but in isolates from different clinical sources. These findings are in agreement with the worldwide spread and upsurge in ESBL-producing *K. pneumoniae* isolates from neonatal sepsis (Khaertynov et al., 2018).

A predominance of *qnr*S followed by *qnr*B *was* observed in our study, which is in agreement with Hammad et al. (2017), who found PMQR genes in all their isolates in a nearby city. Quite surprising was the lack of phenotypic fluoroquinolone resistance despite the presence of PMQR genes, this could be attributed to the silencing of these genes as previously reported by Shetty et al. (2019). Furthermore, it is very important to screen for the presence of these genes as they facilitate the selection of gyrase and topoisomerase genes mutations, resulting in high-level resistance to fluoroquinolones (Rodríguez-Martínez et al., 2016). It is worth noting here that all our isolates harbored also *bla*_NDM_, and the co-existence of isolates harboring the transmissible PMQR genes and *bla*_NDM_ hat the window for therapy choices are gradually declining and these transmissible genes are an imminent threat (Mitra et al., 2019).

There was no correlation between any of the detected resisto-types and the clinical outcome of the infected neonates. It is practical to mention that *K. pneumoniae* typing according to antimicrobial susceptibility profiles was one of the most commonly used approaches in hospital epidemics (Bagattini et al., 2006). However, this typing method has its limitations due to the effect of selective pressure in hospitals on antimicrobial resistance (Christian et al., 2010). In our study, resistotyping alone was not enough for discrimination between different *K. pneumoniae* isolates and thus we used PFGE, which is an established molecular tool for discerning related bacterial strains. Pulsed-field gel electrophoresis affords valuable data to comprehend the epidemiology of MDR *K. pneumoniae* especially carbapenem-resistant isolates and enables the revision and improvement of infection control programs to stop bacterial spread (Mohamed et al., 2017). Pulsed-field gel electrophoresis is considered the most efficient and convenient tool for molecular fingerprinting and characterization of *K. pneumoniae* isolates from either from an outbreak or not when multi-locus sequence typing (MLST) is not obtainable (Han et al., 2013; Zhou et al., 2017).

Molecular categorization using PFGE showed considerable similarity among *K. pneumoniae* isolates and moderate clonal relatedness suggesting the spread of these XDR isolates across the hospital. This cross-contamination might be resulting from hospital staff, infected or colonized patients, and/or contaminated instruments (Celikbilek et al., 2017). There were 14 different pulsotypes among the isolates, which were grouped into four clusters. We found no significant association between different pulsotypes and the existence of various resistance genes. Our findings could be explained by the limited sources of samples, which is in agreement with other studies that found genotypic similarities between various pulsotypes of isolates obtained from limited sources (Christian et al., 2010; Tijet et al., 2014). Furthermore, the lack of genotypic difference between the detected pulsotypes could be attributed to the fact that the most antibiotic-resistant genes are carried on plasmids that are not large enough to be detected in PFGE patterns (Akya et al., 2018).

The results of this study have to be considered in light of the limitations that it is a single-center study of somewhat small sample size. Also, some resistance mechanisms were not evaluated as we focused on the genes for the mainly used antimicrobials in our NICUs.

Notwithstanding these limitations, the study delivers yet unavailable data that can be helpful in both execution of surveillance and spread control of resistance genes, chiefly—but not only—at a local level, to maintain the availability of used therapeutics in treating such infections. Besides, our study is the first to focus on the molecular characterization and the epidemiological transition of XDR *K. pneumoniae* isolated from NICUs in Upper Egypt.

## Conclusion

The development of XDR *K. pneumoniae* is an alarming sign. This mandates the implementation of proper antibiotic stewardship programs with constant surveillance, particularly in units at high risk such as NICUs. Clonal relatedness observed between the isolates suggests necessitating the execution of prevention and management approaches in such hospital settings. Future studies, comprising the assessment of sequence types of these strains by multilocus sequence typing, are justified to better comprehend the transmission, variety, and epidemiology of *K. pneumoniae* in the hospital setting.

## Data Availability Statement

The datasets generated for this study are available on request to the corresponding author.

## Ethics Statement

The studies involving human participants were reviewed and approved by Ethical Committee of the Faculty of Medicine, Minia University. Written informed consent to participate in this study was provided by the participants’ legal guardian/next of kin.

## Author Contributions

NH, RA, and MA conceived and designed the study and wrote the manuscript. NH and RA critically revised the manuscript. All authors carried out the experiments and collected the data.

## Conflict of Interest

The authors declare that the research was conducted in the absence of any commercial or financial relationships that could be construed as a potential conflict of interest.
