# Supplementary material for: Extensively-Drug Resistant Klebsiella pneumoniae Recovered From Neonatal Sepsis Cases From a Major NICU in Egypt
Source: Front Microbiol. 2020 Jun 19;11:1375. doi: 10.3389/fmicb.2020.01375 (PMC7317144; doi:10.3389/fmicb.2020.01375)
Supplement: Supplementary file 1 [file Table_1.DOCX]

Supplementary Material

Table S1: Different isolates with corresponding resisto-type and pulso-type.

| Isolate Number | Resisto-type | Pulso-Type |
| --- | --- | --- |
| KP-1 | B | **P1** |
| KP-2 | A | **P2** |
| KP-3 | A | **P2** |
| KP-4 | A | **P3** |
| KP-5 | A | **P3** |
| KP-6 | B | **P3** |
| KP-7 | A | **P4** |
| KP-8 | C | **P4** |
| KP-9 | A | **P4** |
| KP-10 | D | **P5** |
| KP-11 | A | **P6** |
| KP-12 | A | **P7** |
| KP-13 | A | **P8** |
| KP-14 | B | **P8** |
| KP-15 | A | **P9** |
| KP-16 | A | **P9** |
| KP-17 | A | **P9** |
| KP-18 | A | **P10** |
| KP-19 | E | **P11** |
| KP-20 | B | **P12** |
| KP-21 | A | **P12** |
| KP-22 | B | **P13** |
| KP-23 | B | **P14** |
| KP-24 | F | **P14** |
